# Multiple Supplemental Supernumerary Premolars: Unusual Presentation in a Nonsyndrome Patient

**DOI:** 10.1155/2013/614807

**Published:** 2013-09-09

**Authors:** Ashwini Ramakrishna, Kiran B. Rajashekarappa

**Affiliations:** ^1^Department of Oral Pathology and Microbiology, College of Dental Sciences, Room No. 7, Pavilion Road, Davangere-Karnataka 577004, India; ^2^Sai Multispecialty Dental Clinic, Vidyanagar Main Road, Davangere, Karnataka 577005, India

## Abstract

Supplementary teeth in the dental arch are a rare occurrence. Though they are mostly reported in association with syndromes they can also present in the absence of systemic pathology. This paper reports a case with multiple supernumerary teeth along with discussion of the frequency, types, complications, and management of such occurrence.

## 1. Introduction 

Extra set of teeth in the dental arch are designated as supernumerary which is a well-known dental phenomenon. This dental anomaly is common to primary and permanent dentitions in which the jaw has more teeth than normal. The term supernumerary frequently encompasses the supplementary teeth which are extra teeth that resemble those of the normal series. This developmental anomaly can occur either in the maxilla or in the mandible and is frequently seen in association with syndromes such as Gardner's syndrome, Cleidocranial dysplasia, Trichorhinophalangeal syndrome, and cleft of the lip and palate. They can also be present in the absence of systemic pathology [1].

Prevalence rate of supernumerary teeth for permanent and primary dentitions is said to be between 0.5%–5.3%, and 0.2%–0.8% respectively [2]. However, the prevalence range depends on the methodology for detection and variation in the population. Supernumerary teeth can be unilateral, bilateral, single, or multiple presenting in either of the jaws. There is an apparent predilection of certain sites for supernumeraries and the most common ones are the maxillary mesiodens, followed by supernumeraries of maxillary molar region and those in mandibular premolar region [3]. The supernumerary premolars are mostly seen in the permanent dentition, the prevalence of which is in between 0.075% and 0.26% and they account for only 10% of all the supernumerary cases [2]. Studies on nonsyndrome multiple supernumerary teeth have found 60.9% of the total sample to occur in the mandible and 44.8% in the mandibular premolar region [4].

Morphologically supernumerary teeth are classified into three types, Tuberculate, Supplemental and Conoid forms. Supplemental, tooth is defined as tooth that may resemble a tooth of the normal series [5]. The presence of multiple supernumerary premolars in the absence of any associated syndromes or systemic conditions is relatively rare. This paper describes multiple unilaterally distributed supplemental type of supernumerary premolars of the mandibular arch.

## 2. Case Report

**Figure 1:**
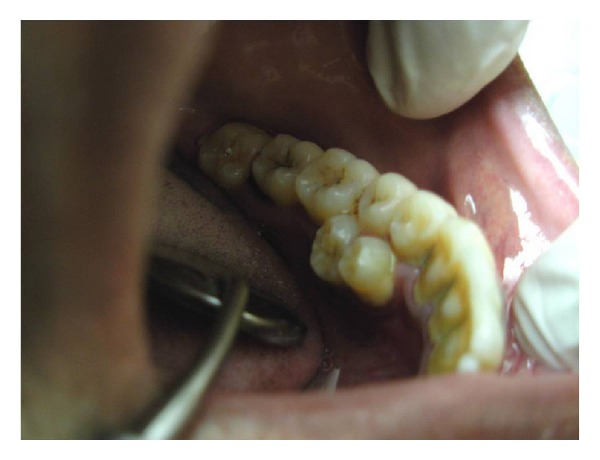
Supernumerary premolars presenting lingual to 34, 35.

A 26-year-old male came to the dental clinic with the chief complaint of pain in the lower left back tooth region. His familial, medical, and dental histories were noncontributory. There was no extra oral deformity in the form of cleft lip or palate and the clavicles were normal. Intraoral examination revealed regular set of permanent dentition along with two completely erupted supernumerary premolars on the left side of the mandibular arch (Figure 1). Both of these teeth presented lingual to the permanent first and second premolars and resembled the permanent premolars in morphology and hence were considered as a supplemental type of supernumerary teeth. Generalized plaque and calculus accumulation was present on the teeth along with generalized gingivitis. 

**Figure 2:**
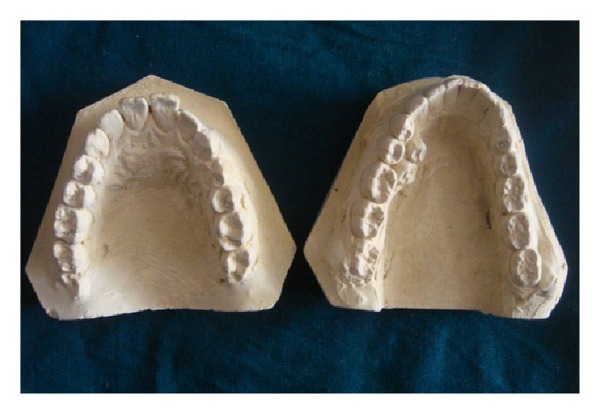
Model cast of maxillary and mandibular arch with the pathology.

Intraoral periapical radiograph (Figure 2) and an orthopantmogram of the patient were taken to evaluate the complete dentition. The radiographs revealed two supernumerary teeth placed lingual to the regular set of mandibular premolars and did not reveal any other impacted or supernumerary teeth or any other pathologies. The supernumerary teeth observed were of normal size and shape with completely formed roots and resembled premolars. 

**Figure 3:**
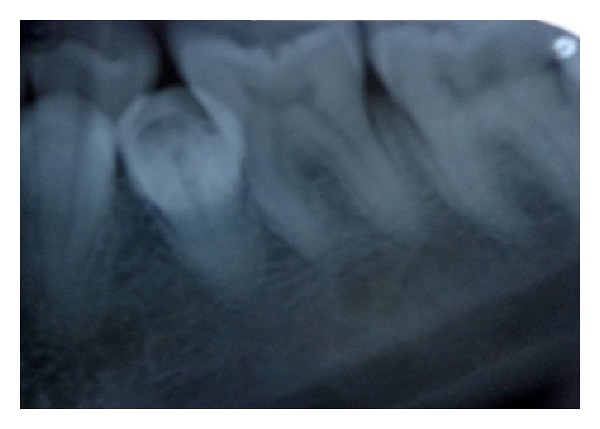
Intraoral periapical radiograph of the supernumerary premolars.

Impressions of upper and lower arches were made and study models were prepared to evaluate the position of the erupted supernumerary teeth and malalignment of the dentition (Figure 3). The patient was informed about the presence of additional number of teeth and was educated about the difficulties associated in maintaining the oral hygiene status and was advised extraction of the same. As family history, medical history, general examination, and extra oral examinations were noncontributory, the diagnosis of nonsyndrome-associated supernumerary was made. Oral hygiene measures were instituted and the follow-up was uneventful.

## 3. Discussion

The presentation of multiple supernumerary teeth in the absence of association with other systemic diseases or syndromes is rare. The prevalence rates of supernumerary premolars are variedly reported in different studies due to the differences in population, age, ethnicity, and applied radiographic techniques [2]. The most common localization for multiple supernumerary teeth is the premolar region (62.1%) and especially that the lower premolar region is a characteristic location for nonsyndrome multiple supernumerary teeth [6]. And the literature states that supernumerary premolars are more frequent in males than females [2]. A majority of supernumerary premolars are of the supplemental type and develop later than their normal counterparts. Patients with a previous history of supernumerary teeth have a 24% possibility of developing single or multiple supernumerary premolars at a later age [7].

Numerous theories have been proposed for the development of supernumerary teeth. The oldest is the theory of Atavism which proposed the development of supernumerary teeth as related to phylogenetic reversion to extinct primates with three pairs of incisors. However, these theories have been discounted now. Two other popularly accepted theories are the Dichotomy theory, which stated that the splitting of the tooth bud into two equal or different-sized parts results in the formation of two teeth of equal size, or one normal and one dysmorphic tooth, respectively [4], and Dental Lamina Hyperactivity theory, which suggests that supernumeraries are found as a result of local, independent, and conditioned hyperactivity of dental lamina [5]. Since individuals with some other dental anomalies and developmental disorders are the ones who frequently present with the supernumerary teeth, combination of hereditary and environmental factors have also been considered in the etiology [6].

Supernumerary teeth can be classified based on morphology as accessory and supplemental and based on syndrome as syndrome associated and nonsyndrome associated [8]. The prevalence for nonsyndrome multiple supernumerary teeth is less than 1%. The male to female ratio has been reported as 9 : 2 [6]. Supernumerary teeth are associated with Gardner's syndrome, Cleidocranial dysplasia, Fabry-Anderson Syndrome, Ehlers-Danlos Syndrome, Down's syndrome, Crouzon's Disease, Hallermann Streiff Syndrome, and Orodigitofacial Dysostosis.

Supernumeraries may erupt normally or may remain impacted or inverted in the jaw or reach heterotopic position or show abnormal eruptive patterns. Various pathological conditions such as delayed eruption or noneruption, displacement of permanent teeth, resorption or malformation of adjacent roots, and cystic formation may be caused because of these teeth [6]. Their occurrence has also been noted in gingiva, soft palate, nasal cavity, maxillary sinus, sphenomaxillary fissure, ophthalmic conchae, maxillary tuberosity, incisive suture, and between the orbit and the brain [9].

Supernumerary teeth are usually asymptomatic and most cases are an incidental finding during a dental visit. Usually if the teeth are asymptomatic, it can be left in place and kept under observation. Surgical removal should be considered based on the pathological sequelae associated with it. And an orthopantomogram should be advised as a part of routine investigation to rule out the presence of multiple supernumerary teeth. In the premolar area, common complications associated with supernumerary teeth are cyst formation (9%) and damage to neighbouring teeth (13%) [8]. Also reports of compression of the supernumerary premolars on the adjacent teeth and their closeness to the mental and inferior dental nerves may lead to pain [2]. Supernumeraries can be associated with other dental anomalies, such as hypodontia, taurodontism, germination, and macrodontia [6]. 

The treatment methods and the timing of surgical removal of supernumerary premolars is much debated among clinicians [10]. Formation of supernumerary premolars are often delayed, and these teeth generally develop on the lingual side of the normal premolars. Developing crypts of the supernumeraries in young patients may be masked by the roots of the normal premolars, which makes early detection on routine radiographs difficult [11]. Hence it is imperative to evaluate the patient clinically and radiologically when a single supernumerary is detected to rule out association with syndromes and for its appropriate and timely management. And it is important on our part to educate the patient about the complications which can arise when the patient is reluctant to the therapy.
